# Differential effects of inbreeding and selection on male reproductive phenotype associated with the colonization and laboratory maintenance of *Anopheles gambiae*

**DOI:** 10.1186/1475-2875-13-19

**Published:** 2014-01-13

**Authors:** Rowida Baeshen, Nkiru E Ekechukwu, Mahamoudou Toure, Doug Paton, Mamadou Coulibaly, Sékou F Traoré, Frédéric Tripet

**Affiliations:** 1 Centre for Applied Entomology and Parasitology, School of Life Sciences, Keele University, Keele, Staffordshire ST5 5BG, UK; 2 Malaria Research and Training Centre, Faculty of Medicine and Dentistry, University of Mali, Bamako, Mali

## Abstract

**Background:**

Effective mating between laboratory-reared males and wild females is paramount to the success of vector control strategies aiming to decrease disease transmission via the release of sterile or genetically modified male mosquitoes. However mosquito colonization and laboratory maintenance have the potential to negatively affect male genotypic and phenotypic quality through inbreeding and selection, which in turn can decrease male mating competitiveness in the field. To date, very little is known about the impact of those evolutionary forces on the reproductive biology of mosquito colonies and how they ultimately affect male reproductive fitness.

**Methods:**

Here several male reproductive physiological traits likely to be affected by inbreeding and selection following colonization and laboratory rearing were examined. Sperm length, and accessory gland and testes size were compared in male progeny from field-collected females and laboratory strains of *Anopheles gambiae sensu stricto* colonized from one to over 25 years ago. These traits were also compared in the parental and sequentially derived, genetically modified strains produced using a two-phase genetic transformation system. Finally, genetic crosses were performed between strains in order to distinguish the effects of inbreeding and selection on reproductive traits.

**Results:**

Sperm length was found to steadily decrease with the age of mosquito colonies but was recovered in refreshed strains and crosses between inbred strains therefore incriminating inbreeding costs. In contrast, testes size progressively increased with colony age, whilst accessory gland size quickly decreased in males from colonies of all ages. The lack of heterosis in response to crossing and strain refreshing in the latter two reproductive traits suggests selection for insectary conditions.

**Conclusions:**

These results show that inbreeding and selection differentially affect reproductive traits in laboratory strains overtime and that heterotic ‘supermales’ could be used to rescue some male reproductive characteristics. Further experiments are needed to establish the exact relationship between sperm length, accessory gland and testes size, and male reproductive success in the laboratory and field settings.

## Background

The fast spread of resistance to insecticides observed in the main malaria vectors, *Anopheles gambiae sensu stricto, Anopheles arabiensis* and *Anopheles funestus *[1,2] suggests that the effectiveness of mass distribution of insecticide-treated nets (ITNs) and large-scale indoor residual spraying (IRS) in reducing the incidence of malaria in endemic countries [3,4] will reach a plateau in the foreseeable future. There is an urgent need for development of not only new chemical compounds, but also of novel and alternative vector control approaches to complement pesticide-based strategies. This urgency explains the renewed interest in vector control using sterile male releases [5] and the rapid expansion of research focused on the release of genetically manipulated mosquitoes unable to transmit malaria [6]. Implicit to these approaches is the necessity to produce large numbers of sexually competitive male mosquitoes from colonized strains in order to target wild vector populations [7,8]. The current knowledge base for mosquito mass-rearing techniques has been accumulated over a number of sterile-male mosquito release programmes attempted during the 1970s [5,7,9]. Although some of those programmes significantly impacted the targeted vector populations, results were generally too poor to warrant their continuation and expansion [5]. These projects generated valuable data about the relative mating competitiveness of laboratory-reared sterile males compared to wild males and putative negative effects of the chemical or radioactivity sterilization steps involved in producing sterile males [5]. They were, however, generally unable to identify the exact genetic and environmental processes associated with colonization and laboratory rearing that negatively affected the reproductive phenotype of mass-produces males [5,8,9].

Colonized strains that are well adapted to the laboratory are able to mate and lay eggs reliably and predictably in the laboratory setting and as such are the starting point of all release control programmes. In the process of establishing a new laboratory colony, the mosquito population undergoes at least one, and possibly several, selective sweeps and genetic bottlenecks as only a fraction of wild captured individuals survive and reproduce in their new environment and the resulting newly colonized strain progressively adapts to the insectary rearing conditions. Therefore, notwithstanding the potential direct negative fitness effects of sterilization or transgenesis [10,11], the genetic changes associated with colonization have the potential to affect the competitiveness and fitness of a candidate release strain [7,8,12]. As an example, the colonization of a wild population of *An. gambiae s.s.* resulted in six-fold decrease in microsatellite allelic richness and two-fold decrease in heterozygosity over a period of two years [13]. Similar patterns have been reported from comparisons of isozyme allelic richness in field population *versus* laboratory strains of *Aedes aegypti* and *Aedes formosus *[14]. Most of the strains used preferentially for genetic engineering of *An. gambiae* have been bred in the laboratory for over 25 years (G3, KIL, etc.) [15] and are most likely to be considerably inbred. Inbreeding is thought to negatively affect fitness by increasing the frequency of homozygotes at the expense of hererozygotes [16]. Negative effects can occur either through the accumulation of deleterious recessive alleles leading to unfit homozygotes - the partial dominance hypothesis, or through the loss of favourable heterozygotes - the overdominance hypothesis [17,18].

The broad causal relationship between inbreeding, decreased phenotypic quality and fitness is well documented from animal breeding studies [19]. In addition, the availability of neutral molecular markers in an increasingly large number of organisms has resulted in a recent flurry of heterozygosity-fitness correlation (HFC) studies reporting correlations between estimates of genetic diversity and fitness components in a variety of wild and captive populations [20]. Currently, none of these studies focus on mosquitoes. However there are some reports of negative effects of inbreeding on the reproductive success of *An. gambiae* laboratory populations (e g, [21]). Moreover, the loss of viability associated with severe inbreeding in attempts to isolate morphological genetic mutants and isogenic lines in Aedine and Anopheline mosquitoes is well documented [14,22].

The expected negative effects of inbreeding on laboratory-reared mosquitoes have led to different schemes for reconstituting their genetic diversity prior to mosquito release programmes [7]. These approaches require crossing and backcrossing laboratory strains with the progeny of field-collected individuals, and are thus not always practical to implement regularly and efficiently [7]. Critically, these schemes ignore the independent contribution of selection for laboratory conditions, another genetic process that could impact the future mating competitiveness of released individuals. Consequently, such schemes can only be considered as hit-or-miss approaches. In addition, there is currently very little understanding of which reproductive traits are negatively impacted by colonization and of how these changes could potentially translate into decreased mating competitiveness in the field [8,23]. Without that knowledge it is virtually impossible to improve on current breeding schemes and laboratory-rearing practices [7].

Here changes in sperm length, testes size and male accessory gland size of *An. gambiae* occurring at different stages of the colonization process were investigated through comparisons of the progeny of field-collected individuals and different laboratory strains aged two to 35+ years. Sperm length has been shown to be very variable in laboratory strains of *An. gambiae *[24] and one study reported that longer sperm were more likely to be stored in the female spermathecae upon mating than shorter ones [25]. There is also limited evidence that sperm length could correlate with male reproductive success in *An. gambiae *[26]. There are currently no studies focusing on variation in testes and male accessory glands size among laboratory or field anopheline populations. In anophelines, the size of both organs is known to increase with male mosquito age and culminate five to six days after emergence [27-29]. Testes size is expected to correlate with the size of the sperm reservoir, and thus could potentially affect the total number of females that males can inseminate. In addition to transferring sperm, male mosquitoes deposit a mating plug in the female atrium during copulation. The mating plug is produced by the male accessory glands and, once deposited in the female, acts as a physical barrier that decreases the likelihood of females mating with other males [21,30]. These plugs also contain an array of sex-peptides that are responsible for inducing a cascade of behavioural changes in females [30-32]. These changes include refractoriness to further mating [30,33,34], host finding, feeding [35], and the initiation of oogenesis [36]. Changes in the size of male accessory glands could affect the size and/or number of plugs that males are able to transfer to females, and therefore determine the number of females they can inseminate.

In addition to comparing those reproductive characters in relation to the age of mosquito colonies, these traits were compared in a colony used to produce two genetically-modified (GM) strains. These strains had been genetically-modified using a two-phase transformation system [37]. The procedure required for genetic transformation leads to two successive genetic bottlenecks that could potentially affect the reproductive phenotype of these and other GM strains created using similar approaches. Finally, we performed crosses between strains and the progeny of field-caught females to create genetically-refreshed outbred strains for comparison with non-refreshed ones. Crosses were also made between old strains to generate heterotic hybrid males. Both types of crosses enabled us to better compare the effects of inbreeding from the effects of selection on the male reproductive phenotype.

This study is the first to describe broad phenotypic changes affecting sperm length, and the size of testes and male accessory glands during the colonization process of laboratory strains of *An. gambiae* and to shed light on the underlying genetic processes leading to these changes. The results have important implications for ecological studies focusing on mosquito reproductive success in the laboratory, as well as for protocols of mass mosquito rearing that are critical to the success of malaria control strategies relying on mosquito releases.

## Methods

### General strain maintenance

All experiments were conducted in 2009 and 2010 in dedicated insectaries of the Centre for Applied Entomology and Parasitology, Keele University. Mosquito strains were kept at 27 ± 2°C, 70 ± 5% relative humidity, with a 12-hr light/dark cycle. Larvae were grown at a density of 200 larvae/l and fed an optimized diet of ground fish food (Tetramin, Tetra, Melle, Germany) [38]. Upon pupation, pupae were transferred to a standard rearing cage made of a 5 L white polypropylene bucket (~20.5 cm height × 20 cm diameter) with a sleeved side opening for introducing and removing mosquitoes and accessories, and the top covered with mosquito netting. Adults were typically maintained at densities of 600–800 adults per enclosure and provided with water and a 5% glucose solution *ad libitum*.

### Molecular form characterization

Field populations of *An. gambiae s.s.* have been subdivided into five chromosomal forms known as Mopti, Savanna, Bamako, Forest and Bissau based on typical arrangements of paracentric inversions located on the 2R chromosome [39-42]. Additionally, two molecular forms exhibiting fixed sequence differences in the intergenic spacer of the ribosomal DNA on the × chromosome and referred to as M and S molecular forms have been identified [43-45]. The M form has recently been elevated to specific status and renamed *Anopheles coluzzii *[46]. Here we will continue to refer to the M and S forms for simplicity. The combination of the ribosomal and inversion polymorphisms currently defines seven cryptic taxa that vary in geographical distribution and habitat use [41]. Consequently, the molecular form of colonized strains varies according to the geographical origin of the wild population they were derived from. Because old laboratory strains may have been contaminated by other strains, prior to conducting this study, all strains were characterized in terms of their molecular form using the PCR/RFLP diagnostic developed by Fanello *et al. *[47].

### Mosquito strains and crosses

#### *Wild-type strains*

The ‘Mopti 2003’ strain, an M-form wild-type strain originally colonized from the village of N’Gabakoro Droit in Mali, West Africa by FT and G Lanzaro was ordered from the MR4 repository. In 2008, a new strain was colonized from the exact same location, which is referred to as ‘Mopti 2008’. The F_1_ progeny of wild-caught females from the same collection site was used for comparison with other colonized strains. Blood-fed females were collected from huts and brought to the insectary at the Malaria Research and Training Centre, Bamako, Mali. Once gravid, females were placed in individual tubes for oviposition. Two days later, individual egg batches and female carcases were shipped to Keele University. DNA extractions from females were carried out immediately using DNAzol (Invitrogen, Carlsbad, CA, USA). The diagnostic PCR/RFLP protocol developed by Fanello *et al. *[47] was used to differentiate *An. gambiae s.s.* females from those belonging the sister species *An. arabiensis*. The same diagnostic also indicated which individuals belonged to the M and S molecular forms among *An. gambiae s.s.* In Mali M-form individuals belong to the Mopti chromosomal form whilst S-form ones can belong to the Savanna or Bamako chromosomal forms [43-45]. Once successfully genotyped, the freshly hatched Mopti M-form *An. gambiae s.s.* broods (1st instar larvae) were placed in trays and reared using a standard larval rearing protocol (see above). Adults were maintained under the same conditions as the other strains until they were dissected for sperm, testes and accessory gland measurements.

Measurements of reproductive traits were also made using two East African wild-type strains colonized in the 1970s. These strains were the Kisumu strain originating from an S-form population from the Kisumu area in Kenya [48], and the KIL strain from Tanzania [15], which was originally an S-form strain but which has since been re-characterized as an M-form because of past contamination with an M-form strain.

#### *Refreshed strains*

To distinguish changes occurring as a result of colony inbreeding from those resulting from selection for laboratory conditions, we also created genetically ‘refreshed’ strains. In the progeny of inbred strains outcrossed to outbred field individuals, reproductive traits negatively affected by inbreeding should be restored whilst character changes resulting from laboratory selection would result in intermediate phenotypes. In 2009, the Mopti 2008 strain was refreshed by crossing 100 F_1_ virgin male progeny from wild blood-fed females collected in huts with 100 virgin female progeny from the colony and *vice versa*. The field-collected females had been sampled from the exact same site used to establish the Mopti 2008 colony. The offspring from these reciprocal crosses were combined to establish a new strain referred to as the ‘Mopti 2008 refreshed 2009’ strain. The Mopti 2003 strain was refreshed in a similar fashion in 2008 leading to the so-called ‘Mopti 2003 refreshed 2008’ strain.

#### *Genetically modified strains*

The EE and EVida3 transgenic strains of *An. gambiae* were used to test whether sequential transgenic modification can affect the male reproductive phenotype. Both strains were derived from the wild-type KIL strain described above using a two-phase targeted genetic transformation system [37]. The Phase 1 EE strain carries a transgene cassette consisting of the phenotypic marker ECFP under the control of the 3xP3 promoter driving its expression in the eyes and other nerve tissues, and the *phiC31* integrase recognition sequence *attP *[46]. The Phase 2 EVida3 strain derived from the EE strain in a second transformation step carries a cassette consisting of 3xP3:ECFP, an additional marker 3xP3:*Ds*Red2 and the synthetic AMP Vida3 sequence with the *An. gambiae carboxypeptidase* promoter, signal peptide and untranslated regions [49]. Both strains were maintained as true-breeding homozygotes.

### Heterotic `supermales’

To further contrast changes occurring as a result of colony inbreeding from those resulting from selection for laboratory conditions, heterotic Kisumu x KIL supermales were created by crossing the old strains with one another. Reproductive traits negatively affected by inbreeding should be fully restored in heterotic males whilst character changes resulting from laboratory selection would result in intermediate phenotypes. For sperm length measurements, 100 virgin Kisumu females and 100 KIL virgin males were combined into one cage and the resulting F_1_ progeny reared to adulthood and dissected for sperm measurements. Because the parental strains differ in their molecular form, the resulting progeny was thus ‘hybrid-like’. The same procedure was used to create heterotic supermales from crosses between KIL females and Mopti 2003 males. The resulting M-form progeny were used for measurements of sperm length and testes and accessory gland size.

### Testes and male accessory glands size

All experiments were made using seven-day old male mosquitoes to ensure that their testes and accessory glands had reached their full size [27,28] and that sperm reservoir contained a large number of mature sperm [29]. Male mosquitoes were killed by freezing and dissected under a trinocular microscope (Leica Microsystems GmbH, Solms, Germany). Fine needles were used to dissect out the testes and male accessory glands. Pieces of cuticle, gut and any detritus were cleared away and the testes and male accessory glands were photographed using a digital still camera (Olympus, Shinjuku, Japan). To determine the size of each testis and accessory glands, their surface on the digital images was calculated with the analysis tools in the ImageJ 1.4 software [50] and converted to mm^2^. All measurements were repeated twice and the average was taken for each sample.

### Sperm length

For sperm measurements, the testes were isolated and transferred to a clean drop of saline. They were then ruptured using fine needles and the liberated spermatazoa were separated from other tissues and collected in the middle of the slide. Slides were immediately examined using differential interference contrast (Nomarski) microscopy (Carl Zeiss AG, Oberkochen, Germany) at 16x magnification. Twenty sperm cells taken from 20 independent fields of view were photographed at 40x magnification using an Infinity X 32 digital camera (Lumenera, Ottawa, Canada) were measured on screen from their digital images using ImageJ and converted to mm. All measurements were repeated twice and were averaged.

### Wing length

Wing length was used as a correlate of mosquito body size [51]. The wings of dissected male mosquitoes were measured from the alular notch to the distal wing margin, excluding the fringe scales, to the nearest 0.01 mm using a binocular microscope with an eyepiece graticule and their length were averaged.

### Statistical analysis

Correlation between sperm length and male body size have been reported elsewhere [26]. Similarly, the calculated surface of testes and accessory glands can be expected to increase with the male body. Mosquitoes from different strains reared under standard conditions can vary in adult body size because of intrinsic and extrinsic reasons and this can potentially confound between-strains differences in reproductive trait sizes. Therefore the full dataset was checked for correlations between sperm length, the size of testes and accessory glands and body size prior to further analyses. Allometric relationships between theses dependent variables and body size were described using the equation log(*y*) = log(*a*) + *b* log(*x*) where the coefficient *b* is the slope of the linear regression but also the power coefficient describing the allometric relationship between the size of the reproductive trait *y* and body size *x* as in *y = kx*^*b*^. When significant, the regression coefficient was used to correct the whole dataset for body size by dividing the variable *y* by *x*^*b*^ (or bodysize^*b*^) prior to further statistical analyses.

All data were checked for deviations from normality and heterogeneity of variances and analysed parametrically or non-parametrically accordingly. All statistical analyses were conducted using the software JMP10 (SAS Institute, Inc).

## Results

### Changes in reproductive phenotype in relation to colonization age

#### *Sperm length*

**Figure 1:**
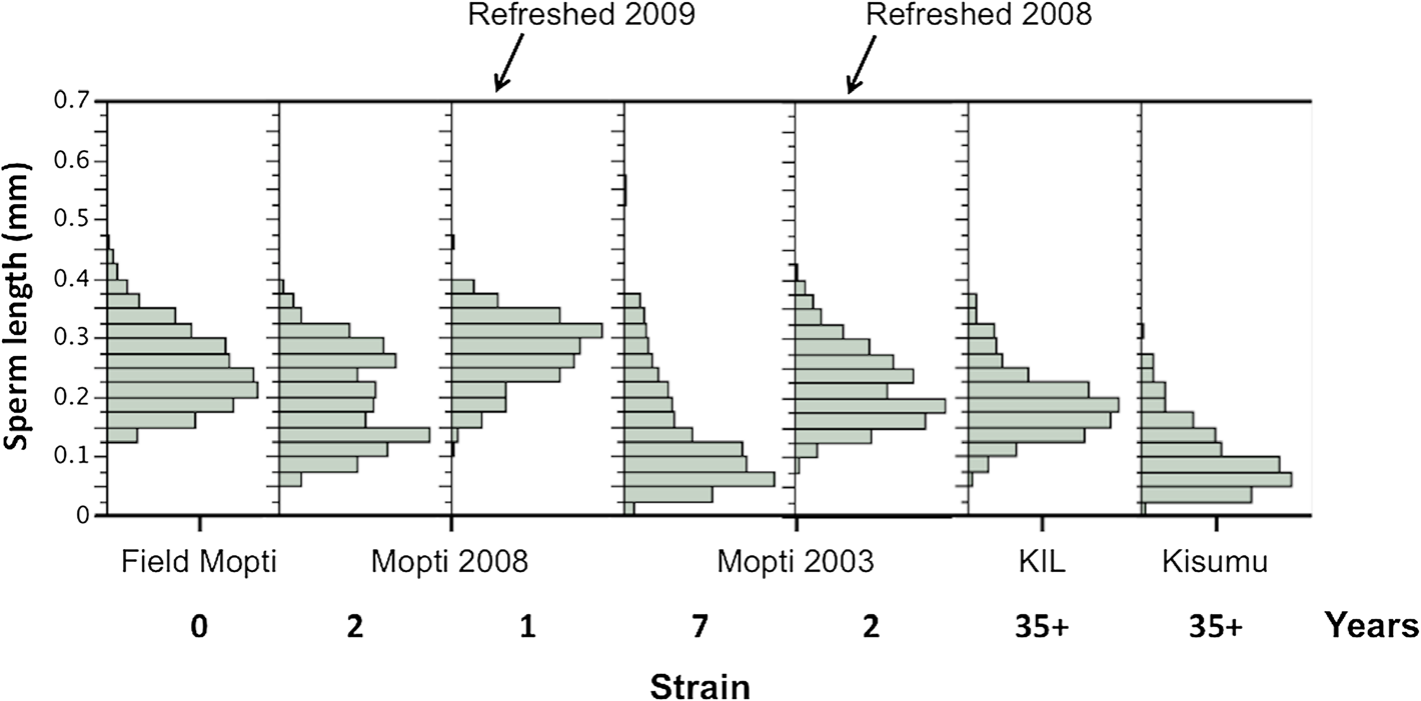
**Distribution of sperm length (mm) in relation to colony age and genetic refreshing.** Twenty mature sperm cells were measured in 134 *An. gambiae s.s.* Field Mopti males and males from the Mopti 2008, Mopti 2008 refreshed 2009, Mopti 2003, Mopti 2003 refreshed 2008, KIL, and Kisumu colonies.

A total of 2,605 sperm cells were measured from 132 individuals from seven strains. Sperm length as compared among the progeny from field-caught Mopti females (Field Mopti) and males from Mopti 2008 and Mopti 2003 strains. All these strains were originally colonized from the same population from the village of N’ Gabacoro Droit, near Bamako, Mali. For these populations, males from older colonies had shorter sperm lengths (Figure 1, Table 1). The mean sperm length of the field Mopti males was 0.250 mm (95% confidence interval = 0.235-0.266 mm) and that of the 35 + -year old Kisumu strain was 0.102 mm (95% CI = 0.089-0.115 mm), resulting in a ~2.5-fold decrease in sperm length over 35 years of laboratory colonization. The two refreshed strains, Mopti 2008 refreshed 2009, and Mopti 2003 refreshed 2008, exhibited sperm length distributions comparable to that of the male progeny of wild females and their distributions clearly shifted towards higher sperm length compared to their non-refreshed counterparts.

**Table 1:** **Characteristics of the sperm length (mm) distribution in male progeny from field-caught females, four strains of contrasted age of colonization and two refreshed strains (see Methods)**

**Strain**	**Age (years)**	**Mean (mm)**	**Median (mm)**	**Range (mm)**
Field Mopti	0	0.251	0.244	0.126–0.462
Mopti 2008	2	0.200	0.195	0.063–0.381
Mopti 2008	1	0.278	0.284	0.120–0.469
refreshed 2009				
Mopti 2003	7	0.139	0.109	0.015–0.557
Mopti 2003	2	0.223	0.213	0.091–0.412
refreshed 2008				
KIL	35+	0.190	0.181	0.054–0.372
Kisumu	35+	0.102	0.087	0.020-0.321

**Figure 2:**
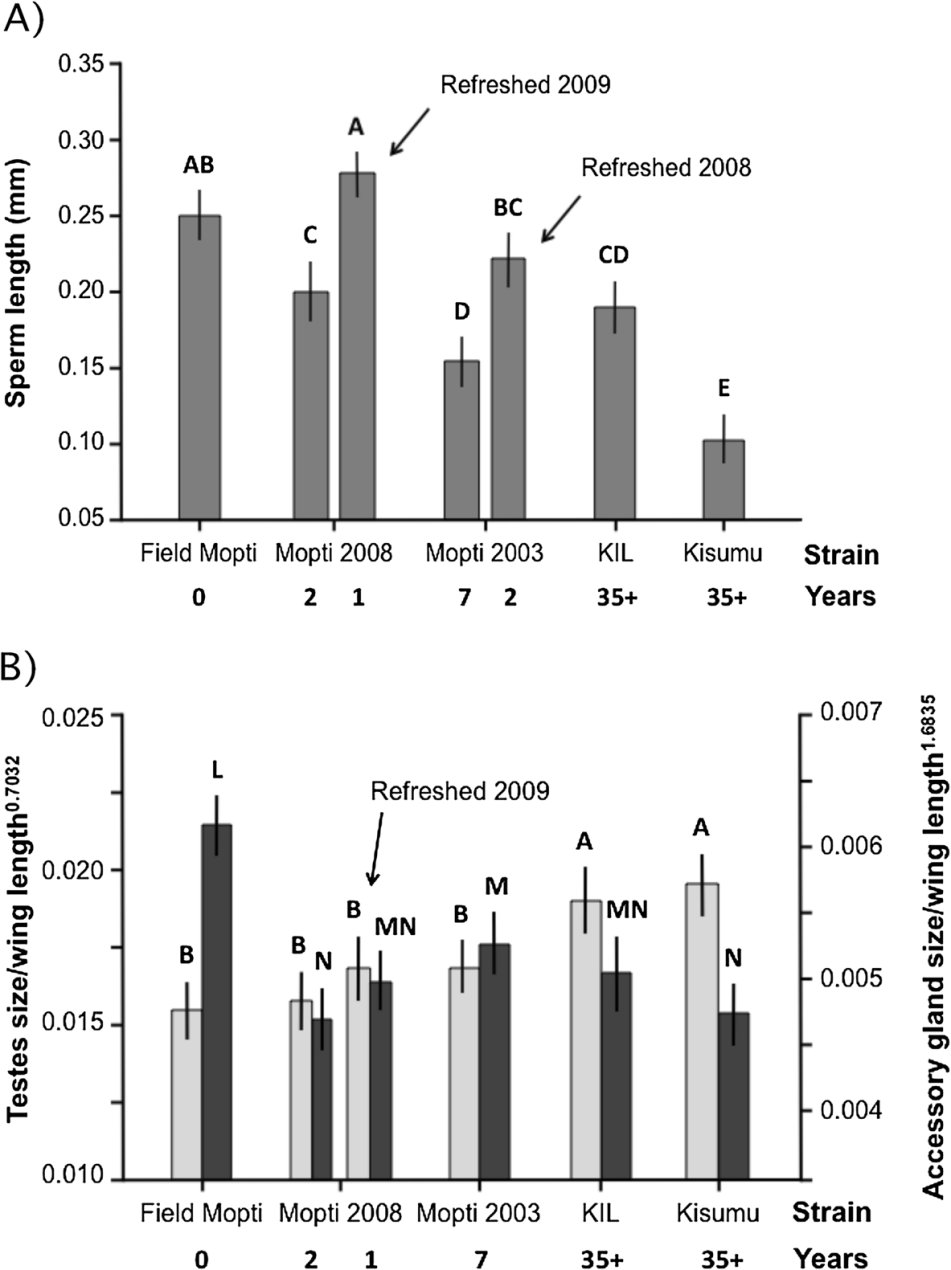
**Mean sperm length and testes and accessory gland sizes in relation to colony age and genetic refreshing. A)** The mean sperm length (20 cells per male) was measured in male progeny of field-collected Mopti, in Mopti strains of various age and in very old strains, and in genetically refreshed colonies. **B)** The mean testes (light grey) and accessory glands (dark grey) size corrected for body size (±95% CI) measured in the same strains. Bars labeled with different letters were significantly different (Tukey *P <* 0.05).

Formal statistical comparisons using nested analysis of variance revealed significant differences among strains and among male individuals nested within strains (ANOVA: strain: *F*_6,2454_ = 418.7, *P <* 0.001; male individual: *F*_124,2454_ = 6.6, *P <* 0.001). Differences among strains were further investigated by an analysis of variance conducted on the mean sperm length per male individual (ANOVA: strain: *F*_6,127_ = 47.4, *P <* 0.001). Post-hoc pair-wise comparisons showed that the grand mean of mean sperm lengths differed significantly between several groups of strains. The Mopti 2008 refreshed 2009, Field Mopti and Mopti 2003 refreshed 2008 had the largest sperm, followed by colonized strains Mopti 2008, KIL, Mopti 2003 and finally the Kisumu strains (Figure 2A).

#### *Testes and accessory gland size*

In contrast to sperm length, the size of testes (corrected for body size) was significantly larger in the old colonized strains KIL and Kisumu strains compared to all other strains (ANOVA: strain: *F*_5,229_ = 14.5, *P <* 0.001; Tukey: *P <* 0.05 in all cases). Field Mopti males had a mean testes size of 0.032 mm^2^ (95% CI = 0.031-0.033) whereas the mean size in males from the old KIL and Kisumu strain was 0.039 mm^2^ (0.037-0.041) and 0.041 mm^2^ (0.038-0.044), respectively, which is roughly equivalent to 1.2 and 1.3-fold increases (Additional file 1). Mean testes size did not differ significantly among field Mopti and the more recently colonized Mopti 2003 and Mopti 2008 strains, and the Mopti 2008 refreshed 2009 strain (Tukey: *P >* 0.05 in all cases) (Figure 2B).

Accessory gland size (corrected for body size) followed a different pattern to that of testes size and decreased significantly between Field Mopti individuals and all colonized strains including the younger ones (ANOVA: strain: *F*_5,232_ = 20.5, *P <* 0.001; Tukey: *P <* 0.05 in all cases). Field Mopti males had accessory glands of mean size 0.036 mm^2^ (0.034-0.038) but the old KIL and Kisumu strains had accessory glands of 0.029 mm^2^ (0.028-0.031) and 0.030 mm^2^ (0.028-0.031), resulting in ~1.2-fold decrease in size. Among colonized strains, males of the Mopti 2003 strain had significantly larger accessory glands than those of the Kisumu and Mopti 2008 strains (Tukey: *P <* 0.05). All other pair-wise comparisons between colonized strains were not significant (Tukey: *P >* 0.05 in all cases) (Figure 2B).

### Changes in reproductive phenotype in relation to genetic transformation

#### *Sperm length*

**Figure 3:**
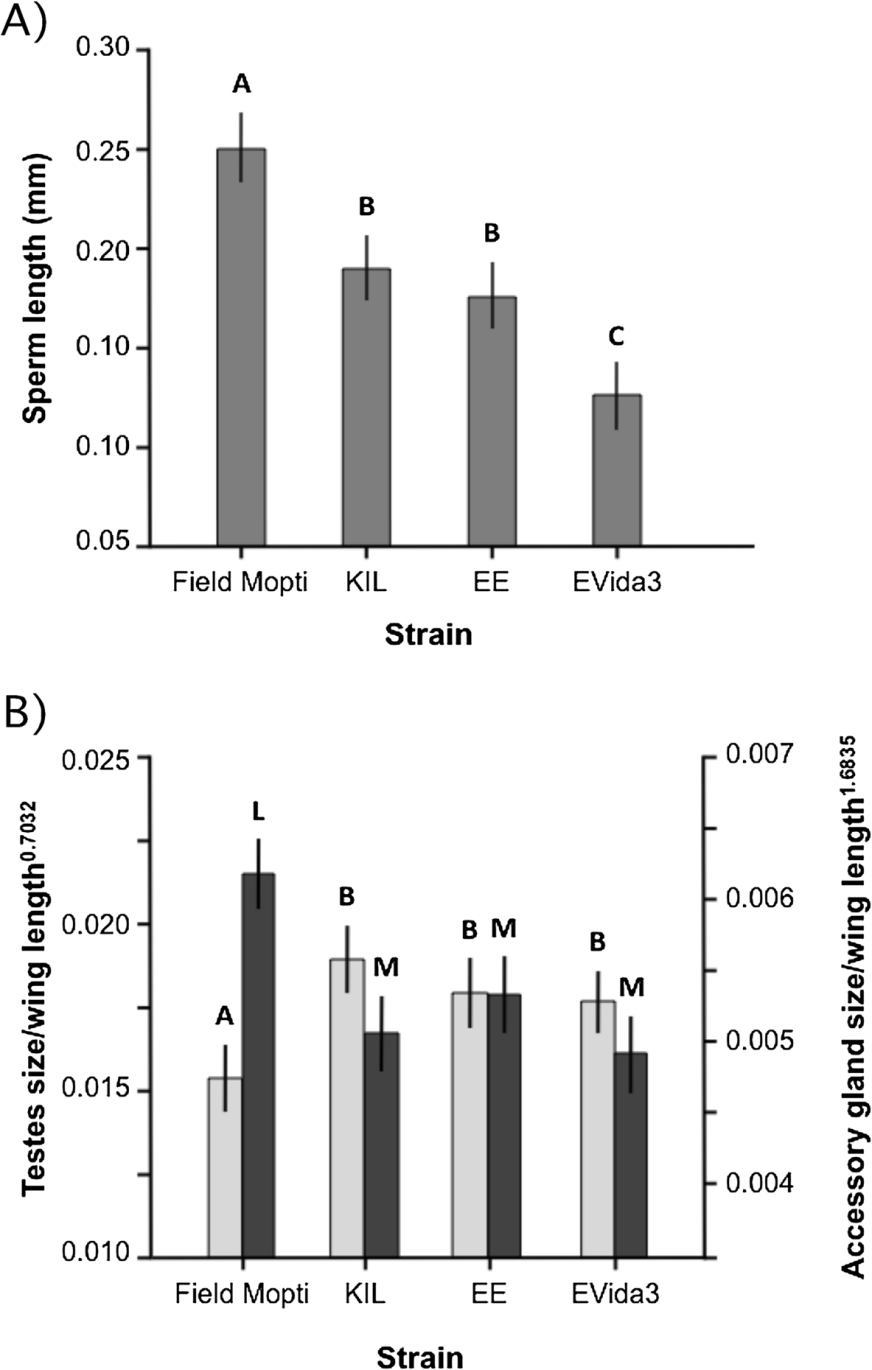
**Mean sperm length and testes and accessory gland sizes in relation to genetic transformation. A)** The mean sperm length (20 cells per male) was measured in male progeny of field-collected Mopti, and the KIL parental line of the transgenic phase-1 EE and EVida3 strains. **B)** The mean testes (light grey) and accessory glands (dark grey ) size corrected for body size (±95% CI) measured in the same strains. Bars labeled with different letters were significantly different (Tukey *P <* 0.05).

Changes in sperm length were also investigated in the KIL strain and the EE and EVida3 strains sequentially derived from KIL using genetic transformation (Figure 3A, Additional file 1). As mentioned above, the 35 + -year-old KIL strain had significantly smaller sperm than Field Mopti males (ANOVA: strain: *F*_3,76_ = 40.3, *P <* 0.001). The phase-I EE GM strain did not show a significant reduction in sperm length compared to the wild-type KIL strain from which it is derived. However the phase-II EVida3 strain, itself derived from the EE line through an additional step of genetic modification, had significantly reduced sperm size compared to the KIL and EE strains (Tukey: *P <* 0.05) (Figure 3A). The mean sperm length of the EVida3 was almost half (47%) as long as that of Field Mopti males.

#### *Testes and accessory gland size*

Whilst the Field Mopti strain had significantly smaller testes (ANOVA: *F*_3,151_: 12.4, *P <* 0.001) and larger accessory glands (ANOVA: *F*_3,152_: 21.7, *P <* 0.001) than the KIL, EE and EVida3 strains, there were no significant differences in testes size and accessory gland size between males of the KIL strain and the two GM strains derived from it (Tukey: *P >* 0.05 in all cases) (Figure 3B, Additional file 1).

### Changes in reproductive phenotype in relation to heterosis

#### *Sperm length*

**Figure 4:**
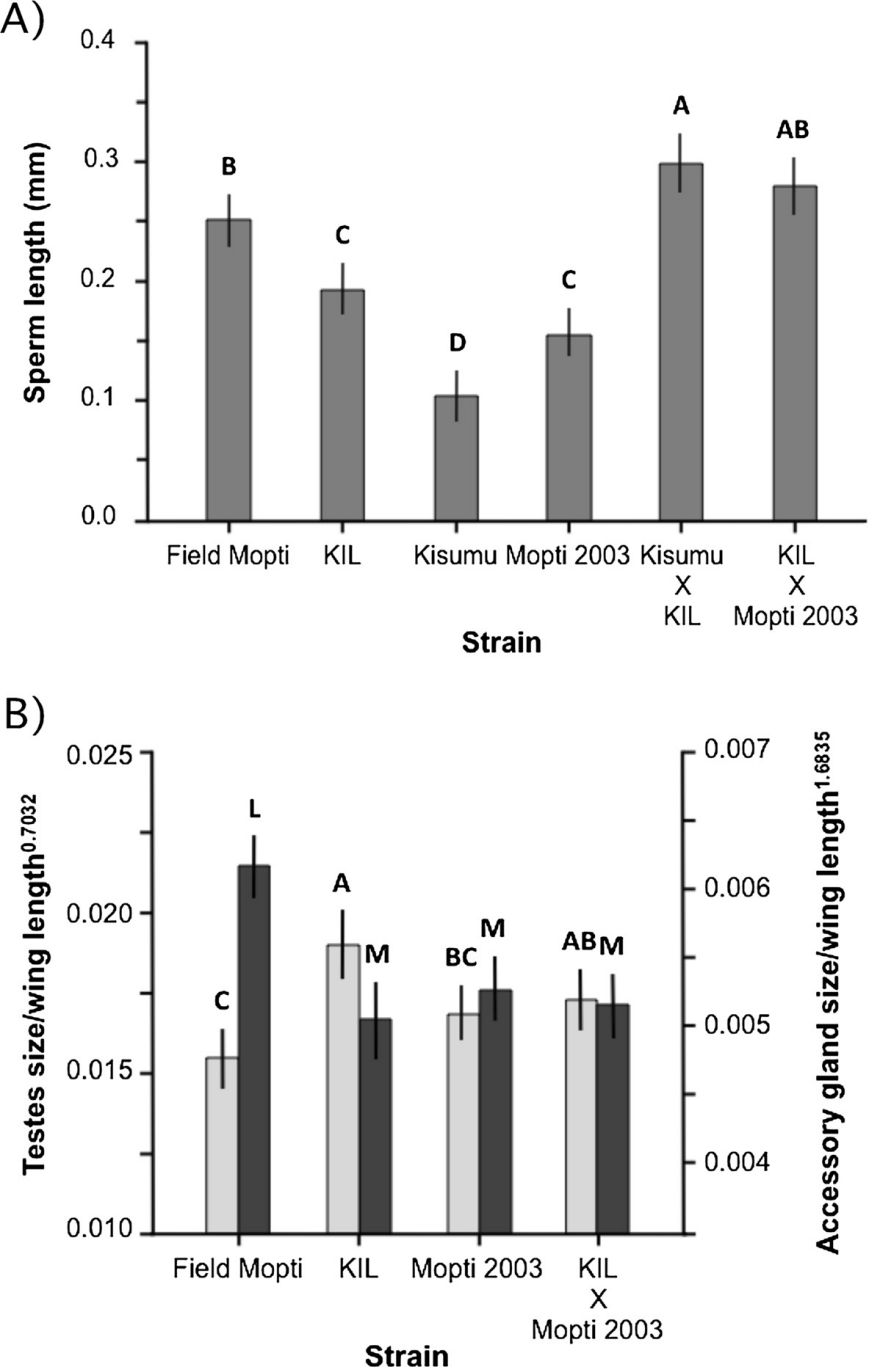
**Mean sperm length and testes and accessory gland sizes in relation to heterosis. A)** The mean sperm length (20 cells per male) was measured in male progeny of field-collected Mopti, the older KIL, Kisumu and Mopti 2003 strains and heterotic males crosses between the later strains. **B)** The mean testes (light grey) and accessory glands (dark grey) size corrected by wing length^2^ (±95% CI) measured in the same strains. Bars labeled with different letters were significantly different (Tukey *P <* 0.05).

The three oldest strains (Mopti 2003, KIL, and Kisumu) were used to create heterotic hybrid males and their sperm were compared to males of the parental strains and Field Mopti males. There were large differences in mean sperm length between the older inbred lines and their heterotic male progeny (ANOVA: strain: *F*_5,109_ = 52.5, *P <* 0.001) (Figure 4A, Additional file 1). The progeny of Kisumu females crossed with KIL males had significantly longer sperm than all old strains (Tukey: *P <* 0.05 in all cases). The sperm from that cross was nearly three times longer than that of the Kisumu males but did not significantly differ in length from that of the second cross. Heterotic males produced by crossing KIL females with Mopti 2003 males had much longer sperm than either parental strains (*P <* 0.05) but not significantly longer than that of Field Mopti males (*P >* 0.05) (Figure 4A, Additional file 1).

#### *Testes and accessory gland size*

Comparisons of testes and accessory gland size were also made between heterotic males produced by crossing KIL females with Mopti 2003 males. The resulting progeny exhibited testes larger than Field Mopti males (ANOVA: *F*_3,153_ = 10.0, *P <* 0.001; Tukey: *P <* 0.05) but intermediate and not significantly different from either of that of its parental strains (*P >* 0.05) (Figure 4B). Similarly, their accessory gland were significantly smaller than that of the male progeny of field individuals (ANOVA: *F*_3,157_ = 18.6, *P <* 0.001; Tukey: *P <* 0.05) but not significantly different from that of the two parental strains (*P >* 0.05) (Figure 4B).

#### *Relationship between reproductive traits and body size*

**Figure 5:**
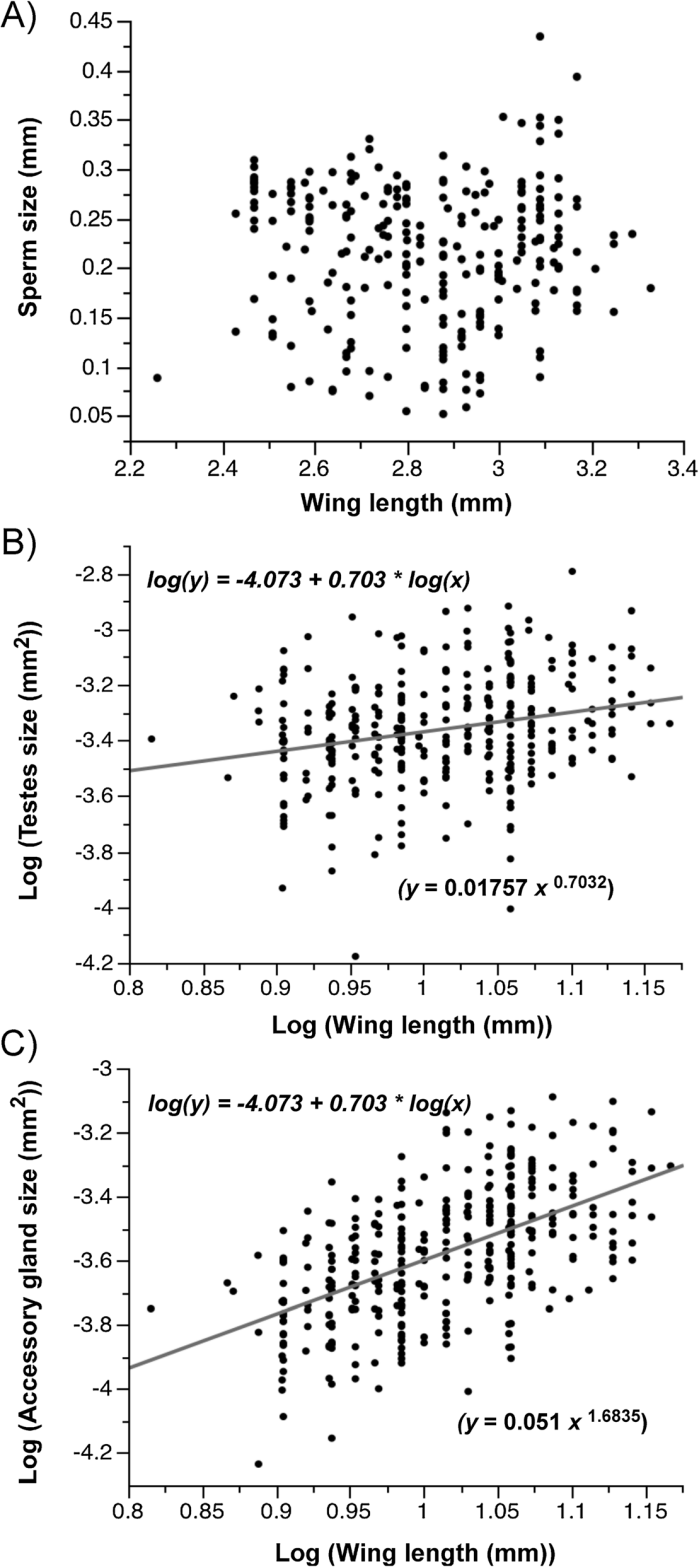
**Relationship between male body size and reproductive traits. A)** The mean sperm length (20 cells per male), and **B)** mean testes and **C)** accessory gland sizes are shown in relation to male body size measured as wing length. Equations of significant allometric relationships are indicated in their linear and exponential form (in brackets). There was no significant allometric relationship between mean sperm length and male body size.

The relationship between body size and the uncorrected data (Additional file 1) of sperm length, testes size, and accessory gland size was explored using the regression log(*y*) = log(*a*) + *b* log(*x*) where the coefficient *b* is the slope of the linear regression and the power coefficient of the allometry between reproductive trait and body size (see Methods). Surprisingly, no positive linear relationship was found between sperm length and male body size across all strains (regression: *n =* 208, *T =* -0.97, *P =* 0.333) or within any of the strains (*P >* 0.05 in all cases) (Figure 5A). The same was true when examining the relationship between body size and the minimum, maximum and median sperm length of male mosquitoes across all strains (*P >* 0.279 in all cases). Within strains, a relationship between minimum sperm length and body size was only found in the Mopti 2008 strain (*P =* 0.017) but this was not supported in the 10 other strains (*P >* 0.05 in all cases). No significant relationship was found between the median and maximum sperm length and body size within any of the strains (*P >* 0.05 in all cases).

Across all strains there was significant relationship between log (male body size) and log (testes size) (regression: *n =* 352, *T =* 4.8, *P <* 0.001) (Figure 5B). However, the relationship did not hold when looking at each strain (*P >* 0.05). Furthermore when combining the potential effects of strain and body size in an analysis of covariance, the relationship between log (testes) and log (body size) was not significant, thereby indicating that it was mostly caused by variation in male body size between strains (ANCOVA: strain: *F*_*8,342*_*=* 9.4, *P <* 0.001; log(wing length): *F*_*1,342*_*=* 1.1, *P =* 0.297).

There was a significant positive linear relationship between the log (accessory gland size) and log (body size) (regression: *n =* 354, *T =* 12.71, *P <* 0.001) (Figure 5C) and the same was true when correcting for strain effects thereby indicating that the relationship was not caused by variation in body size between strains (ANCOVA: strain: *F*_*8,354*_*=* 12.4, *P <* 0.001; log(wing length): *F*_*1,354*_*=* 121.5, *P <* 0.001). The relationship between log (accessory gland size) and log (male body size) was also found to be significant in seven out of nine strains (*P <* 0.05 in all cases).

## Discussion

This is the first study to examine evolutionary changes in reproductive traits following colonization and adaptation to the laboratory environment in *An. gambiae* and to distinguish the effects of different evolutionary forces acting on its reproductive phenotype. Contrasting changes were observed in the length of sperm, and the size of testes and male accessory gland in relation to the age of mosquito colonies. Laboratory mosquitoes generally had increasingly larger testes but shorter sperm and smaller accessory glands than their wild-type counterparts. Sperm length decreased with time of colonization. Comparisons among genetically transformed, genetically refreshed, and in heterotic males supported the idea that this decrease in male sperm length was due to inbreeding. In contrast to that pattern, testes size was found to increase over time and was larger in the long-established KIL and KIS strains, suggesting progressive adaptation to laboratory conditions. Furthermore, testes size did not differ between the KIL strain and the derived transgenic EE and Evida3 lines, suggesting that this change was driven by laboratory selection rather than by the two sequential genetic bottlenecks associated with the two-phase genetic transformation system. In addition testes size was not recovered with strain refreshment or in heterotic males confirming that this change was driven by laboratory selection. Finally, the size of male accessory glands decreased over time following a trajectory opposite to that of testes, albeit at a much faster rate, suggesting that selection for laboratory conditions led to a quick decrease of this organ’s size. Here again, further comparisons of these organs in relation to genetic transformation suggested that accessory gland size did not change in relation to genetic bottlenecking. In addition, accessory gland size was not improved in refreshed strains and in heterotic males thereby supporting the idea that adaptation to laboratory conditions drove these size change rather than inbreeding.

These results are important because they are the first to clearly highlight significant morphological differences between laboratory strains and wild mosquitoes and therefore serve to emphasize the need to validate laboratory findings with semi-field or field studies particularly when focusing on mosquito mating ecology. Although the exact relationship between the size of these male traits and mating success and fecundity was not demonstrated here, there is evidence from previous studies suggesting that changes in sperm, testes and accessory glands may affect male fitness (see below). Thus these changes have the potential to affect their mating competitiveness in the context of sterile or GM mosquito releases. Furthermore, whilst the negative effects of inbreeding on sperm size was counteracted in males from refreshed strains and heterotic males, strain refreshment was not sufficient to restore the wild-type-like testes and accessory gland phenotype. This suggests that producing males with a mating phenotype comparable to that of wild males might require complex breeding and rearing scheme. The possibility of creating heterotic ‘supermales’ with enhanced mating performance from old inbred lines adapted to the laboratory is an exciting development that may be an effective way for producing large numbers of competitive males. This exciting discovery warrants further evaluation.

That sperm length progressively decreases with colonization time, hence inbreeding, suggests that it could be used as a practical biomarker for describing levels of inbreeding in mosquito colonies. This constitutes a substantial improvement over measures of inbreeding relying on molecular markers heterozygosity since correlations between heterozygosity at neutral markers and fitness are notoriously weak [20,52]. The unreliability of inbreeding estimates based on molecular markers is further compounded by their sensitivity to demographic events commonly affecting mosquito colonies, such as contaminations with other strains that can occur unbeknown to mosquito colony users. Thus sperm length comparisons between laboratory strains and between these strains and wild individuals from their population of origin provide a simpler way of comparing levels of inbreeding than the comparatively time consuming and expensive molecular approaches.

Currently the exact relationship between sperm length and male mosquito fitness is unknown and further studies are underway to establish that causal link. In anopheline mosquitoes, some studies suggest that larger sperm have a higher likelihood of fertilizing the eggs. A comparative study of *Anopheles quadriannulatus*, *Anopheles darlingi* and *An. gambiae s.s.* revealed high degrees of sperm length polymorphism in males from these four taxa [24]. It is noteworthy that for *An. gambiae* the size reported in that study ranged from 0.026-0.100 mm which is smaller than the range of the most inbred KIL (0.054-0.372 mm) and Kisumu strains (0.020-0.321 mm) [24]. Interestingly, the same study [24] and a study of *An. arabiensis *[25] showed that sperm recovered in the female spermathecae were comparatively larger than those measured from testes suggesting that larger sperm have the highest likelihood of fertilizing the eggs than smaller ones [24,25]. In the outbred Keele strain, average sperm length was found to be ~0.199 mm (range 0.100-0.250 mm) and negatively genetically correlated with oviposition success [26]. These results are not necessarily incompatible with the patterns of sperm length in relation to inbreeding reported here and would suggest that the Keele strain with its intermediate sperm length was indeed not strongly inbred at the time of that study [26]. The same study showed that there was significant intra-specific variation in sperm length among males from the same *Anopheles* species as shown here. The exact function of sperm polymorphism in *An. gambiae* is currently still unknown and, despite the strong effect of inbreeding observed in this study, it is noteworthy that strains of increasing age retained comparable levels of sperm length variation despite a constant decrease in mean size and a shift towards higher proportions of small sperm. As outlined elsewhere [26], sperm variation could simply be maintained because of natural variation in the size of female sperm storage organs. Comparative studies in anopheline species [24] and stalk-eyed flies [53] suggest that sperm length and the female spermatheca size broadly co-evolve. Within species, experiments in *Drosophila *[54] and dung beetles [55] showed that the competitiveness of different-size sperm depended on the size of the female sperm storage organs. Taken together, these findings suggest that optimal sperm length could vary with spermatheca size, which strongly correlates with female body size [56]. Because female size depends on the female larval growth conditions [51] having polymorphic sperm might allow males to have higher reproductive success across a wide range of female body and spermatheca sizes.

The changes observed in testes and accessory gland size in relation to colonization time can be explained by the unique mating conditions associated with insectary rearing. In natural populations males await females in male-dominated swarms thereby creating conditions in which male competition for females is high and reproductive success may largely be driven by female choice [57]. This type of conditions, which bear analogies with leks, typically leads to very skewed distributions of male reproductive success with males of higher phenotypic quality securing most copula [57,58]. The 50:50 sex ratio artificially created by combining distinct cohorts of freshly hatched female and male imagoes in small laboratory cages results in starkly different selection pressures on males. Anopheline males can typically inseminate up to five females per night [59]. Given the large number of virgin females available to males in crowded cages, the best males cannot possibly secure all mating, hence there may be more mating opportunities for males of lesser phenotypic quality. Male reproductive success may then depend less on the male phenotypic quality and female choice than on the male capacity to inseminate as many females as possible in a short window of time. In other words, laboratory rearing leads to increased sperm competition and larger testes size may be strongly selected for as they enable more frequent mating. The relationship between testes size and sperm competition is well described across a large number of taxa including insects [60-62]. Experimental evolution studies have also shown an increase in testes size in relation to increased sperm competition in *Drosophila *[63] and the bruchid beetle *Callosobruchus maculatus *[62].

Positive selection for testes size could result in negative selection on male accessory gland size if there is a negative genetic correlation between these two traits. Such a negative correlation could exist if, for example, there is a trade-off between sperm and sex-peptide production. However, the decrease in male accessory gland size over time appears to have been quicker than that of testes size. In anophelines the size of accessory gland is highly dependent on male mating status and decreases following mating [28,29,64]. The mating plug produced by the accessory glands is thought to acts as a physical barrier to further mating [21,30]. It is noteworthy that *An. gambiae* males are thought capable of inseminating up to five females per night but of producing only two full mating plugs [64]. If cage rearing leads to scramble competition for females the importance of securing as many copula as possible might outweigh that of preventing females from further mating through the physical barrier of a full plug. Alternatively, female fecundity might not depend on a full mating plug under insectary conditions. Plugs contain sex-peptides that are responsible for inducing a number of behavioural changes [30-32] such as refractoriness to further mating [30,33,34], host finding and feeding [35], and the initiation of oogenesis [36]. They also contain the vitellogenic steroid hormone 20-hydroxyecdysone that may be an important determinant of female fecundity [65]. Thus the adaptive reduction in plug size observed in colonized strains could be linked to one or several changes in the female traits that are mediated by plug composition. Detailed analyses of changes in plug composition following colonization would therefore be required in order to delineate which of these is driving the observed changes in accessory gland size.

This study found no correlation between the mean sperm length and the body size of males in any of the strains studied or across all strains. In a study on the Keele strain of *An. gambiae*, the two traits were significantly correlated in some but not all datasets [26]. Here, there was an overall significant linear relationship between body size and testes size across all strains but the relationship did not hold when correcting for strain effects. In contrast, accessory gland size strongly correlated with body size across and within strains.

## Conclusions

This study highlights adaptive and non-adaptive changes affecting laboratory-reared mosquito populations. Although rearing protocols may vary across laboratories and institutions, one can expect that the adaptive increase in testes size and decrease in accessory gland size observed here across several strains will apply to most if not all laboratory colonies. In addition, negative inbreeding effects are expected to accumulate as mosquito colonies age and these changes may affect their reproductive phenotypes as highlighted by the continuous changes observed in sperm length in relation to colonization time.

These findings emphasize the limitations of laboratory-based studies focusing on the mating process and reproductive success of *An. gambiae s.s.* and invite particular caution when extrapolating those findings to wild mosquito populations. Additionally, mass-rearing programmes gearing-up for the release of sterile or genetically modified males should as much as possible create environmental conditions that create field-like sexual selection and sperm selection pressures on males in order to insure their mating performance.

## Competing interests

The authors declare that they have no competing interests.

## Authors’ contributions

RB, NE, FT, MC, SFT planned the experiments. RB, NEE, DP, MT conducted the experiments. RB and FT analysed the data. RB and FT wrote the manuscript. All authors read and approved the final manuscript.

## Supplementary Material

Additional file 1 **Mean (±95% CIs) sperm length and testes and accessory gland size.** Reproductive traits were measured in male progeny from field-caught females and males from four strains with contrasted age of colonization, two refreshed strains, the EE and EVida3 genetically modified strains, as well as the heterotic male progeny of the Kisumu x KIL and KIL x Mopti 2003 crosses. Click here for file
