# Supplementary material for: Differential effects of inbreeding and selection on male reproductive phenotype associated with the colonization and laboratory maintenance of Anopheles gambiae
Source: Malar J. 2014 Jan 13;13:19. doi: 10.1186/1475-2875-13-19 (PMC3896703; doi:10.1186/1475-2875-13-19)
Supplement: Additional file 1 — Mean (±95% CIs) sperm length and testes and accessory gland size. Reproductive traits were measured in male progeny from field-caught females and males from four strains with contrasted age of colonization, two refreshed strains, the EE and EVida3 genetically modified strains, as well as the heterotic male progeny of the Kisumu x KIL and KIL x Mopti 2003 crosses. [file 1475-2875-13-19-S1.docx]

**Additional file 1 Mean (±95% CIs) sperm length and testes and accessory gland size**

| **Strains** | **Sperm length (mm)** | | **Testes surface (mm^2^)** | | **Accessory gland surface (mm^2^)** | |
| --- | --- | --- | --- | --- | --- | --- |
| **Various ages and refreshed** | *N*^†^ | Mean^§^ | *N* | Mean | *N* | Mean |
| Field Mopti | 20 (397) | 0.250 (0.235-0.266) | 39 | 0.032 (0.031-0.033) | 42 | 0.036 (0.034-0.038) |
| Mopti 2008 | 14 (280) | 0.199 (0.176-0.223) | 40 | 0.031 (0.029-0.033) | 39 | 0.024 (0.023-0.026) |
| Mopti 2008 refreshed 2009 | 20 (390) | 0.278 (0.267-0.288) | 39 | 0.033 (0.032-.0035) | 39 | 0.026 (0.025-0.027) |
| Mopti 2003 | 20 (382) | 0.155 (0.122-0.187) | 37 | 0.034 (0.032-0.036) | 39 | 0.028 (0.027-0.029) |
| Mopti 2003 refreshed 2008 | 20 (397) | 0.221 (0.206-0.236) | - | - | - | - |
| KIL | 20 (399) | 0.190 (0.178-0.201) | 40 | 0.039 (0.037-0.041) | 39 | 0.029 (0.028-0.031) |
| Kisumu | 20 (390) | 0.102 (0.089-0.115) | 41 | 0.041 (0.038-0.044) | 40 | 0.030 (0.028-0.031) |
| **Genetically modified** |  |  |  |  |  |  |
| EE | 20 (386) | 0.175 (0.164-0.186) | 35 | 0.036 (0.034-0.038) | 37 | 0.029 (0.027-0.031) |
| EVida3 | 20 (396) | 0.125 (0.089-0.151) | 41 | 0.036 (0.034-0.038) | 38 | 0.028 (0.026-0.029) |
| **Heterotic males** |  |  |  |  |  |  |
| Kisumu x KIL | 17 (339) | 0.297 (0.259-0.335) | - | - | - | - |
| KIL x Mopti 2003 | 18 (360) | 0.277 (0.268-0.285) | 41 | 0.034 (0.032-0.036) | 41 | 0.026 (0.025-0.027) |

**^†^**Twenty sperm were measured from ~20 individuals per strain. Sample sizes are number of individuals and total number of sperm measured per strain (in brackets).

^§^The grand mean of mean sperm length per individuals is reported here.
